# Computing Thermal Half-Lives: Machine-Learned Design Rules for Arylazopyrazole Photoswitches

**DOI:** 10.1021/acscentsci.6c01356

**Published:** 2026-08-21

**Authors:** Xuanchi Yu, Tao Li

**Affiliations:** School of Chemistry and Chemical Engineering, State Key Laboratory of Synergistic Chem-Bio Synthesis, Shanghai Key Laboratory of Electrical Insulation and Thermal Aging, 12474 Shanghai Jiao Tong University, Shanghai 200240, China

## Abstract

Machine-learned interatomic potentials enable large-scale prediction of arylazopyrazole thermal half-lives and mechanisms.

## Graphical abstract

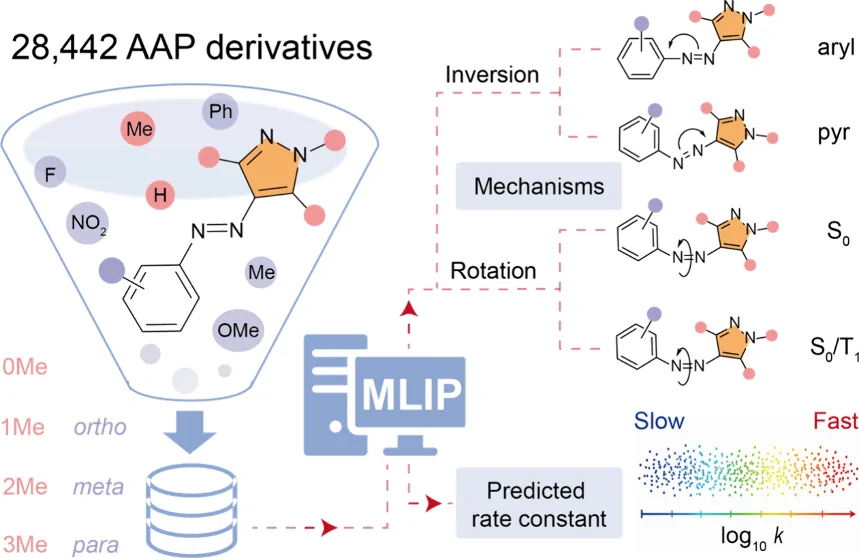

For azo compounds, an important class of molecular photoswitches, the thermal half-life is a central design parameter because it determines how long the metastable state persists and whether a photoswitch is suited to a particular application. Since the seminal report of arylazopyrazole (AAP) as a photoswitch with exceptionally long thermal half-life (∼1000 d) in 2014,^1^ extensive structural diversification of this scaffold has been pursued. However, the incorporation of a pyrazole ring introduces additional asymmetry and multiple methylation patterns, further complicating the relationship between molecular structure and thermal half-life. In a recent issue of *ACS Central Science*, González, Dietschreit, and co-workers employed a machine-learned interatomic potential (MLIP) trained on spin-flip time-dependent density functional theory (TD-DFT) to map the thermal isomerization landscapes of 28,442 arylazopyrazole derivatives and predict their relaxation rates at scale (Figure 1).^2^

**Figure 1:**
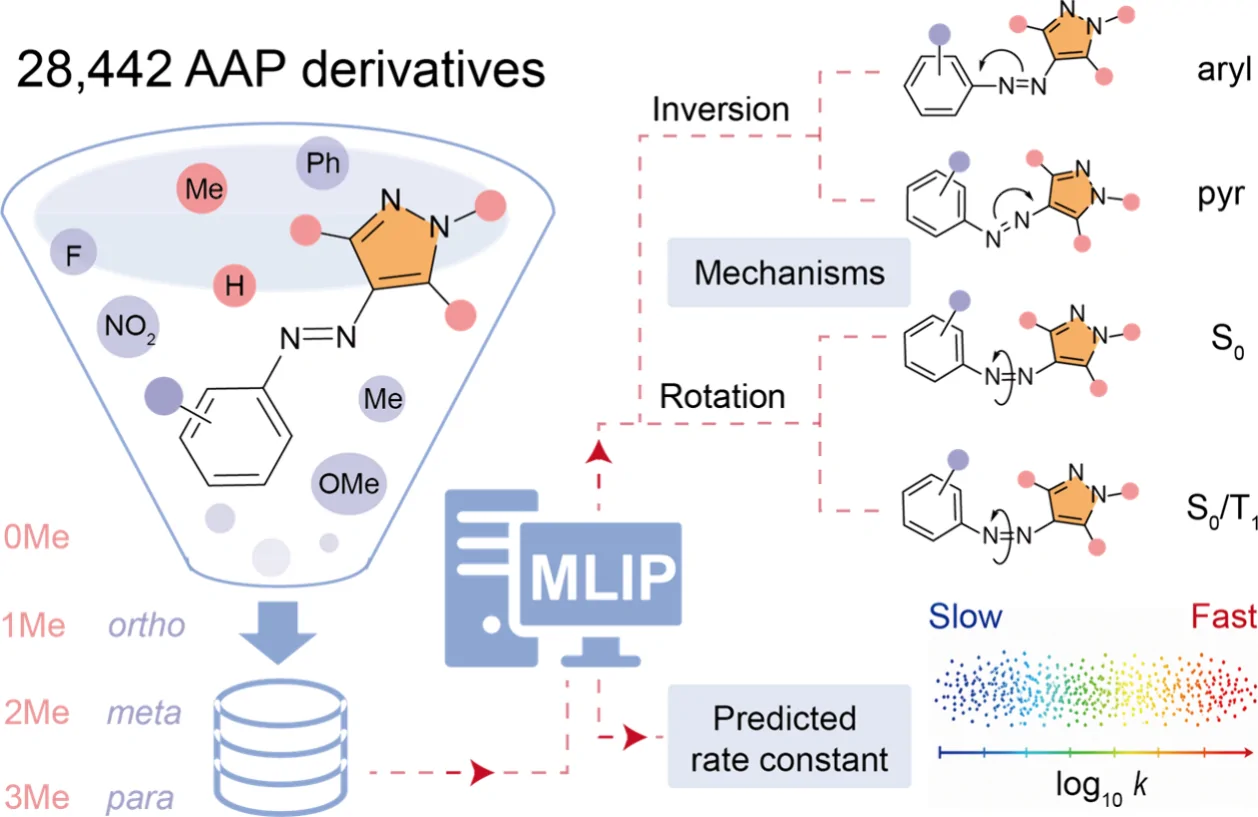
Schematic overview of the MLIP workflow for mapping thermal relaxation mechanisms and rate constants across AAP chemical space.

Previous experimental studies have begun to establish structure–half-life relationships in arylazopyrazoles.^3–5^ For arylazo-1,3,5-trimethylpyrazoles (3Me-AAPs), all examined *para*-substituents shortened the thermal half-life relative to the unsubstituted parent, whereas *meta*-substitution generally exerted little influence. *ortho-*Substitution produced substantially more complex effects because of the combined influence of electronic effects and steric interactions. Additionally, 3Me-AAPs generally exhibit faster thermal *Z*→*E* isomerization than 1Me-AAPs (only the pyrazole nitrogen is methylated), resulting in shorter half-lives. This acceleration has been attributed to changes in the relative stabilization of the *Z*–isomer and transition state (TS).^6^ In 1Me-AAPs, the T-shaped *Z*–isomer is stabilized by an intramolecular C–H···π interaction. Further methylation disrupts this interaction while providing additional dispersion stabilization to the TS, thereby lowering the thermal isomerization barrier. However, exhaustive synthesis and characterization of accessible derivatives are impractical, leaving open the question of whether experimentally derived trends represent general design principles or merely local patterns within the limited chemical space explored.

Here, MLIPs offer a decisive advantage by enabling the systematic exploration of chemical space that would otherwise require extensive compound-by-compound synthesis and characterization. Their broader value lies in shifting photoswitch development from synthesis-led trial and error toward computation-guided prioritization – a “compute first, synthesize second” strategy

(Figure 2a). Computational prescreening of absorption spectra using DFT or TD-DFT is already common in photoswitch development.^7–9^ Reliable prediction of thermal lifetimes, however, has remained considerably more challenging because thermal relaxation rates are exponentially sensitive to activation barriers and may arise from several competing pathways. Encouragingly, the workflow developed by González, Dietschreit, and co-workers showed notable agreement with experimental data for 15 *para*-substituted 3Me-AAPs:^5^ nine predicted rates deviated by less than 1 order of magnitude, with one additional prediction falling within 2 orders of magnitude. This agreement is encouraging for an automated high-throughput TS theory workflow.

**Figure 2:**
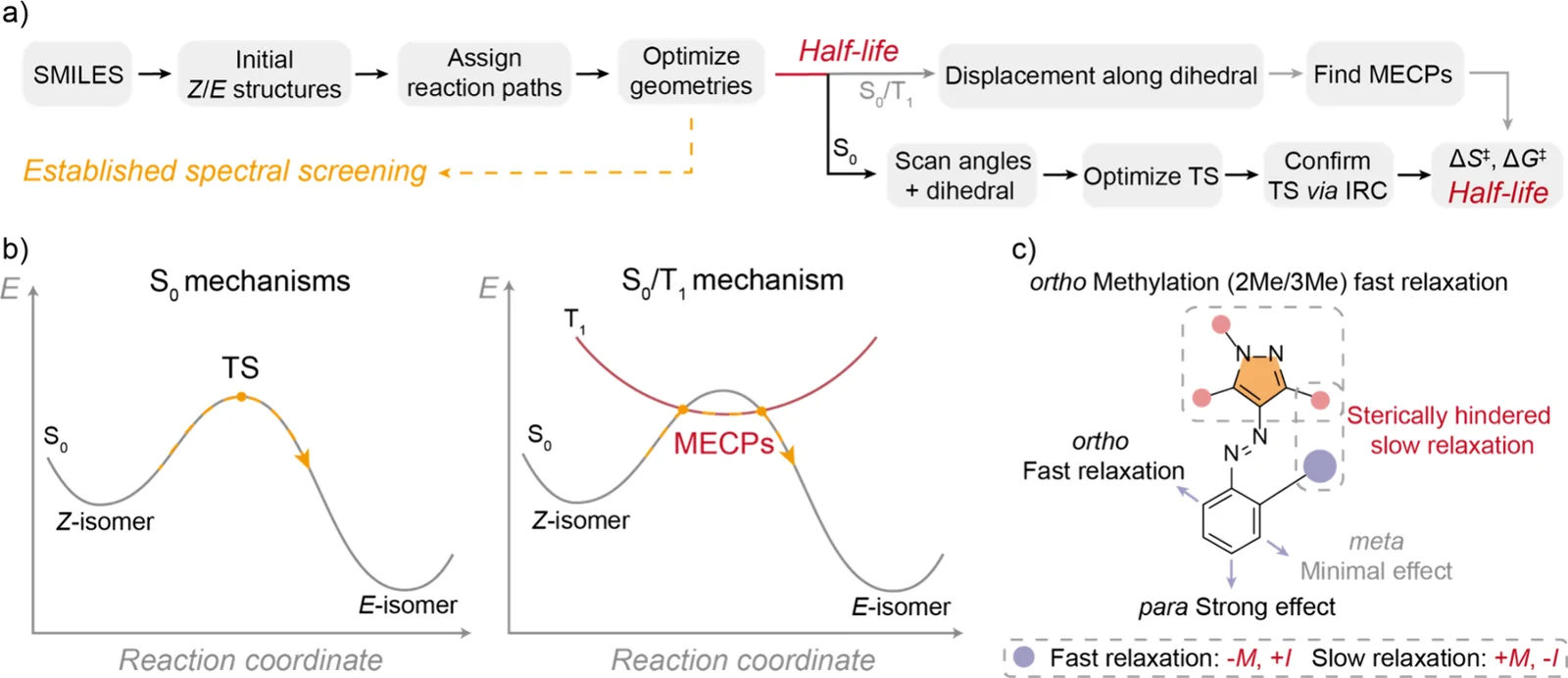
a) Toward a “compute first, synthesize second” strategy by integrating established spectral screening with MLIP-based thermal rate prediction. b) Schematic potential energy profiles illustrating the thermal ground-state rotational pathway (left) and the nonadiabatic rotational pathway (right) involving the lowest triplet state T_1_ (red surface). Orange circles denote TS and minimum energy crossing points (MECPs). c) MLIP-derived design rules for AAPs.

More importantly, the workflow independently locates and evaluates four competing thermal isomerization pathways for each compound, including two inversion pathways, a ground-state rotation pathway, and a nonadiabatic rotation pathway mediated by intersystem crossing (ISC) to the lowest triplet state T_1_ (Figure 2b). It thereby moves beyond analyses centered on a single presumed dominant pathway. Crucially, the overall relaxation rate is obtained by summing the contributions from all accessible pathways rather than selecting only the lowest barrier route. The resulting distribution analysis establishes a central mechanistic conclusion: AAP thermal relaxation is intrinsically multichannel. Specifically, methylation at the pyrazole carbon (adjacent to the azo group) generally favors faster thermal relaxation (Figure 2c). However, when bulky *ortho*-substituents are introduced in the aryl ring simultaneously, excessive steric congestion can restrict the geometrical motions required for inversion and rotation pathways, yielding derivatives with unexpectedly long thermal half-lives. On the aryl side, the enrichment analysis identifies a clear statistical bias: electron-withdrawing character (−M) or electron-donating inductive effect (+I) substituents, particularly at the *ortho* position, are preferentially associated with the fast relaxation regime, whereas electron-withdrawing inductive effect (−I) and electron-donating character (+M) substituents occur more frequently among slow relaxing AAPs.

At the level of substitution position, the authors found that *ortho*- and *para*-substitution strongly modulate thermal relaxation, whereas *meta*-substitution has little influence. The statistical analysis further associates *ortho*-substitution predominantly with faster relaxation, although some studies indicate that this effect remains strongly substituent-dependent, as shown by *ortho*-difluorinated 1Me-AAP, for which a thermal half-life of 46 years has been reported.^4^ Nevertheless, population level analysis remains highly informative, revealing broad statistical regularities that are difficult to identify from individual molecular series. The mechanism enrichment analysis further suggests that +M substituents favor rotation over inversion and preferentially promote the triplet-mediated rotational pathway. In contrast, −M substituents are more strongly associated with inversion or ground-state rotation.

Taken together, these findings show that structural modification does not simply raise or lower a single thermal isomerization barrier. It also redistributes the competition among multiple pathways and thereby controls both thermal relaxation rates and the dominant relaxation mechanism.

Altogether, the MLIP framework developed by González, Dietschreit, and co-workers marks a significant advance toward computation-guided photoswitch design. By combining large-scale rate prediction with mechanism-resolved design rules, the framework turns thermal relaxation rate from a property measured primarily after synthesis into a variable that can guide candidate selection beforehand. Combined with established computational screening of spectral properties, this approach points toward an integrated “compute first, synthesize second” strategy for photoswitch discovery. This capability extends the impact of the workflow beyond the photoswitch community, offering materials scientists and medicinal chemists a rational route to identifying azo units with thermal half-lives tailored to specific photoresponsive functions.
